# Supplementary figures and images for: Adulteration of beeswax: A first nationwide survey from Belgium
Source: PLoS One. 2021 Sep 9;16(9):e0252806. doi: 10.1371/journal.pone.0252806 (PMC8428765; doi:10.1371/journal.pone.0252806)

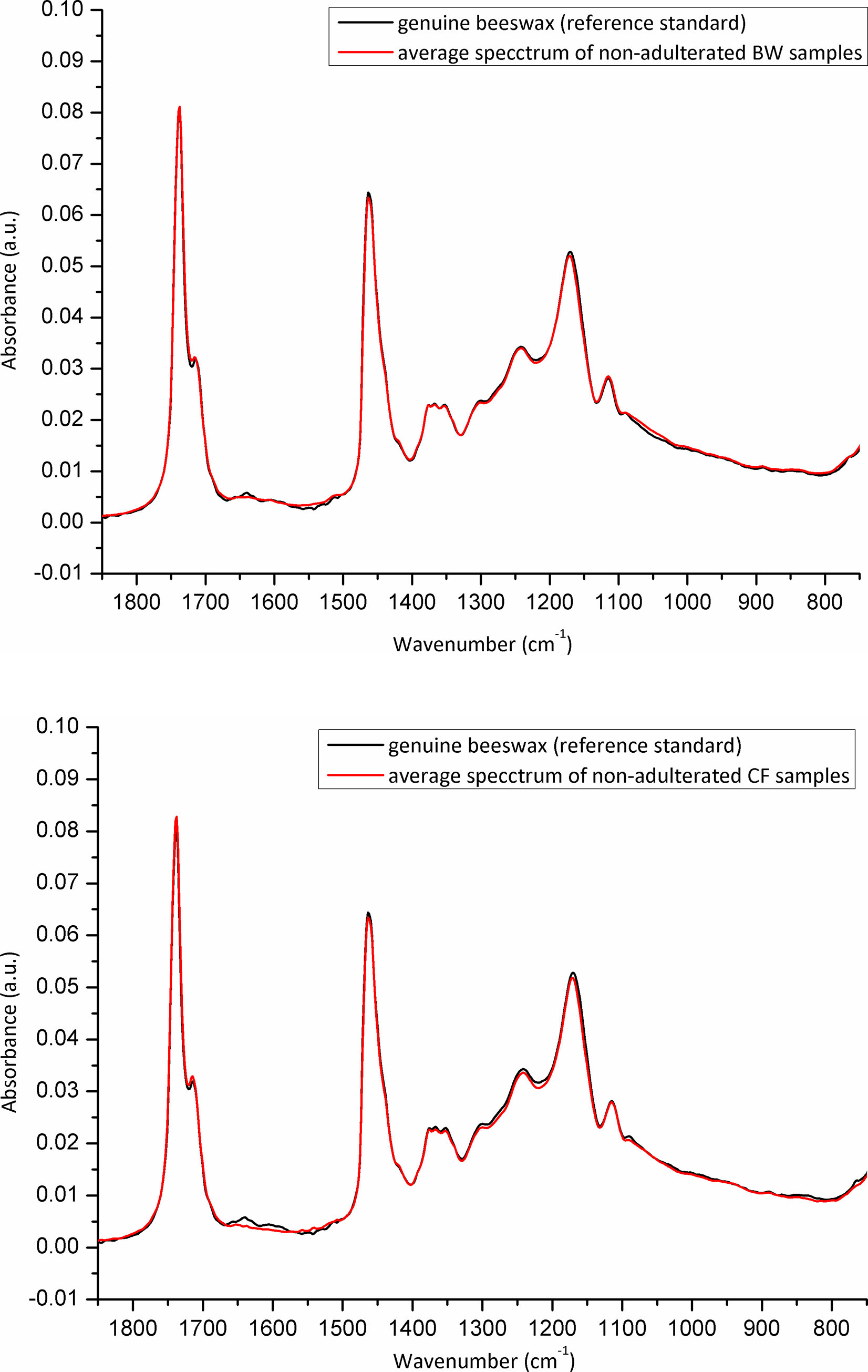

Supplement: S2 Fig — Comparative spectral features of: An average spectrum of non-adulterated beeswax samples (N = 88) versus genuine beeswax (reference standard) [A] an average spectrum of non-adulterated comb foundation samples (N = 6) versus genuine beeswax (reference standard) [B]. Wavenumber, the number of waves per unit distance; cm, centimetre; a.u. is for the absorbance unit. (TIF) [file pone.0252806.s002.tif]
